# Suspicion de candidoses superficielles à Libreville: profils cliniques et distribution des espèces de *Candida* spp. (2022-2024)

**DOI:** 10.48327/mtsi.v6i3.2026.742

**Published:** 2026-07-27

**Authors:** Coella Joyce MIHINDOU, Reinne MOUTONGO MOUANDZA, Bridy Chesly MOUTOMBI DITOMBI, Ardin Dimitri MOUSSAVOU MABICKA, Charleine MANOMBA BOULINGUI, Christian MAYANDZA, Roger Hadry SIBI MATOTOU, Noe Patrick M’BONDOUKWE, Magalie ESSOMEYO NGUE MEBALE, Denise Patricia MAWILI MBOUMBA, Marielle Karine BOUYOU AKOTET, Maryvonne KOMBILA†

**Affiliations:** 1 Departement de parasitologie-mycologie et de medecine tropicale (DPMMT), Universite des sciences de la sante (USS), Libreville, Gabon; 2 Cabinet medical d’Oloumi (CMO), Libreville, Gabon; † *In memoriam*

**Keywords:** Candidoses superficielles, Siège, Prévalence, Gabon, Afrique subsaharienne, Superficial candidiasis, Location, Prevalence, Gabon, Sub-Saharan Africa

## Abstract

**Contexte:**

La prise en charge des lésions évocatrices des mycoses superficielles repose encore souvent sur des traitements empiriques, favorisant l’usage inapproprié des antifongiques et l’émergence de résistance. L’objectif de cette étude était de déterminer la prévalence et le profil clinique et épidémiologique des candidoses dans une population de patients ayant bénéficié d’un diagnostic biologique à Libreville.

**Méthode:**

Les données des patients ayant bénéficié d’un examen mycologique complet devant une lésion suspecte de mycose superficielle, de 2022 à 2024, au cabinet médical d’Oloumi ont été recueillies et analysées au sein du département de parasitologie-mycologie et de médecine tropicale de l’Université des sciences de la santé. Les variables sociodémographiques, cliniques et mycologiques ont été comparées à l’aide du logiciel Statview 5.0.

**Résultats:**

Au total, les données de 1256 patients ont été analysées. La prévalence de *Candida* spp. était de 18,4 %. Les onyxis candidosiques des mains (54,5 %), des pieds (11,3 %) et les lésions inguino-crurales (11,3 %) étaient les formes cliniques majoritaires. *Candida albicans* était l’espèce la plus fréquente (61 %) et majoritairement isolée chez les hommes (82,1 %; n = 46/56), tandis que les espèces non *albicans* avec comme chef de file, *C. parapsilosis* (41,7 %) prédominaient chez les femmes (37,8 %; n = 74/169) (p < 0,01). Cependant, les espèces non *albicans* colonisaient majoritairement les ongles et plus spécifiquement ceux des pieds (p < 0,01) avec comme espèces responsables *C. parapsilosis* (24,2 %), *C. guillermondii* (5,2 %) et *C. tropicalis* (3,5 %).

**Conclusion:**

Les champignons levuriformes, notamment les *Candida* spp., sont des étiologies fréquentes des mycoses cutanées probables à Libreville. Bien que *C. albicans* soit l’espèce prédominante, il existe une prévalence non négligeable des lésions cutanées associées à des espèces non *albicans* notamment *C. parapsilosis*. Les atteintes des ongles et des grands plis sont plus fréquemment d’origine candidosique.

## Abstract (en)

**Background:**

The management of lesions suggestive of superficial fungal infections often still relies on empirical treatments, which can lead to the inappropriate use of antifungals and the emergence of resistance. This study aimed to determine the prevalence of candidiasis and its clinical and epidemiological profile in a population of patients who underwent laboratory diagnosis in Libreville.

**Methods:**

Data on patients undergoing comprehensive mycological examinations for lesions suspected to be superficial fungal infections between 2022 and 2024 at the Oloumi Medical Clinic were collected and analyzed within the Department of Parasitology, Mycology and Tropical Medicine at the University of Health Sciences. Sociodemographic, clinical and mycological variables were compared using StatView 5.0 software.

**Results:**

A total of 1,256 patient records were analyzed. The prevalence of *Candida* spp. was 18.4%. The most common clinical forms were candidal onychomycosis of the hands (54.5%), feet (11.3%), and inguinal-crural lesions (11.3%). *Candida albicans* was the most prevalent species (61%), predominantly isolated in men (82.1%; n = 46/56). In contrast, non-*albicans* species, led by *C. parapsilosis* (41.7%), predominated in women (37.8%; n = 74/169) (p < 0.01). Non-*albicans* species, however, predominantly colonized the nails, specifically those of the feet (p < 0.01), with the causative species being *C. parapsilosis* (24.2%), *C. guillermondii* (5.2%), and *C. tropicalis* (3.5%).

**Conclusion:**

Yeast-like fungi, particularly *Candida* spp., are common causes of probable cutaneous fungal infections in Libreville. Although *C. albicans* is the predominant species, there is a significant prevalence of skin lesions associated with non-*albicans* species, particularly *C. parapsilosis*. Lesions affecting the nails and major skin folds are more frequently caused.

## Introduction

L’incidence croissante des infections causées par des agents pathogènes fongiques humains constitue une menace croissante et sous-estimée, particulièrement en raison de la résistance accrue aux traitements antifongiques. Les infections fongiques superficielles comptent parmi les plus courantes dans le monde, touchant environ 20 à 25 % de la population [9,17,32]. Outre les derma-tophytoses et les malassezioses, les candidose*s* superficielles constituent les étiologies les plus évoquées par les cliniciens [33].

Les levures du genre *Candida* appartiennent à la flore commensale humaine et peuvent coloniser les muqueuses des voies génitales, urinaires, respiratoires et gastro-intestinales, ainsi que la cavité buccale, les ongles, le cuir chevelu et la peau [2,10]. Bien que *Candida albicans* soit l’espèce la plus fréquemment isolée, les espèces de *C.* non *albicans*, telles que *C. parapsilosis*, *C. krusei* et *C. glabrata* sont de plus en plus fréquemment responsables d’infections superficielles [6,16,19,28].

Au Gabon, les lésions cliniques superficielles représentent les circonstances de diagnostic les plus fréquentes dans les demandes adressées au laboratoire de mycologie du département de parasitologie-mycologie et de médecine tropicale (DPMMT) de la Faculté de médecine de l’Université des sciences de la santé (USS). Il s’agit du laboratoire de référence pour le diagnostic clinique et biologique des affections mycosiques au Gabon. Cependant, peu d’informations sont disponibles pour la communauté médicale sur le spectre clinique et la diversité des espèces responsables, alors que les moyens thérapeutiques restent limités. En effet, des prévalences élevées de résistance de *C. albicans* et des *C.* non *albicans* aux antifongiques usuels tels que le fluconazole, l’éconazole, l’amphotéricine B ont été observées [24,30]. La prescription empirique des antifongiques (notamment le fluconazole, molécule la plus accessible au Gabon), chez les patients immunodéprimés pour la prévention des infections mycosiques opportunistes, ou chez les immunocompétents pour le traitement curatif présomptif, est un facteur de risque d’émergence des résistances à cette molécule.

Une analyse récente des données colligées du DPMMT et de celles des patients vivant avec le VIH (PvVIH) rapporte une modification du profil de la flore fongique des candidoses digestives à Libreville [26,30,38]. Cependant, l’épidémiologie des candidoses superficielles reste peu connue. Générer des informations sur ces pathologies courantes permettrait l’élaboration d’algorithmes décisionnels et de protocoles de prise en charge adéquate, et constituerait un plaidoyer auprès des décideurs politiques pour une meilleure accessi-bilité à d’autres antifongiques azolés.

La présente étude a pour objectif de décrire la prévalence, le profil clinique et la diversité fongique des candidoses superficielles dans une population de patients porteurs de lésions cliniques à Libreville.

## Méthodes

Il s’agit d’une étude rétrospective descriptive menée dans une structure sanitaire de Libreville, le cabinet médical d’Oloumi (CMO) par l’équipe du DPMMT de l’USS.

Les données des registres des patients qui avaient bénéficié d’un examen mycologique complet devant une lésion suspecte de mycose superficielle de 2022 à 2024 ont servi de matériel d’étude. Les informations suivantes ont été reportées sur une fiche de recueil de données et analysées au sein du DPMMT: présence d’une lésion suspecte de mycose, site de la lésion, résultat de l’examen mycologique, âge et sexe. Selon leur âge, les patients ont été répartis en quatre groupes: les nourrissons ayant moins de 2 ans, les enfants âgés de 2 à 17 ans, les adultes âgés de 18 à 59 ans et les seniors ayant au moins 60 ans.

Les prélèvements ont été réalisés en respectant les conditions d’asepsie. Les lésions situées au niveau de la peau, des muqueuses, des ongles et du cuir chevelu ont été prélevées par raclage des squames sur le bord des lésions (peau), par frottement à l’aide d’un écouvillon stérile ou grattage au moyen d’un bistouri stérile (ongle), par prélèvement des squames du cuir chevelu et des poils ou cheveux atteints. Les prélèvements vaginaux ou des urines ont été effectués en cas de suspicion de candidoses des muqueuses génitales.

L’examen microscopique direct a été réalisé entre lame et lamelle avec l’ajout d’une goutte de solution d’hydroxyde de potassium à 30 % pour éclaircir les préparations épaisses telles que celles des phanères. Une solution physiologique saline a été utilisée pour les écouvillons.

La culture a été effectuée par ensemencement sur les milieux usuels de mycologie (Sabouraud-chloramphénicol et Sabouraud-chloramphénicol-actidione), suivie d’une incubation à 37 °C. Les cultures ont été contrôlées après 24 heures et quotidiennement pendant 8 jours. Une culture était considérée comme positive en présence de colonies crémeuses, bombées, de couleur blanche à crème. Pour le prélèvement des muqueuses, lorsque *C. albicans* était identifiée, la présence d’au moins 10 colonies permettaient d’affirmer le diagnostic de candidose.

L’identification et la différenciation entre *C. albicans* et *C. dubliniensis* ont été effectuées à l’aide des milieux chromogènes Candida ID2 (Biomérieux), selon les recommandations du fabricant et les procédures décrites dans la littérature [12]. L’identification a été complétée par la réalisation de l’auxanogramme à l’aide de galeries API20 C AUX [3,35].

La candidose superficielle était définie par l’association d’arguments cliniques et mycologiques, reposant sur: (i) la présence d’une lésion cutanéo-muqueuse évocatrice de mycose superficielle; (ii) un examen mycologique direct positif mettant en évidence des levures bourgeonnantes et/ou des pseudofilaments, associés à une réaction inflammatoire locale attestée par la présence de leucocytes (≥10 cellules par champ) et/ou (iii) une culture positive avec une croissance ≥10 colonies de *Candida* spp.

Dans cette étude, le diagnostic de candidose reposait exclusivement sur les critères cliniques et mycologiques. La confirmation biologique au laboratoire constituait l’élément décisionnel pour l’indication d’un traitement antifongique, et non sur la réponse au traitement, conformément aux recommandations internationales [5,34].

La réponse au traitement relevait de l’évaluation de la prise en charge et n’a pas été utilisée comme critère de confirmation diagnostique.

La colonisation par *Candida* spp. était définie par son isolement en culture chez des sujets asymptomatiques, en l’absence de signes cli-niques évocateurs et de critères microscopiques d’invasion [5,31,34].

Les données ont été reportées sur un fichier Excel et analysées grâce au logiciel Statview 5.0. Les données qualitatives ont été présentées sous formes de proportions et comparées grâce au test du chi2 ou au test Exact de Fisher. Le seuil de significativité a été considéré pour un p < 0,05.

## Résultats

Les données de 1256 patients ayant bénéficié d’un examen de mycologie pour suspicion de mycose cutanéomuqueuse ont pu être analysées. Le sexe féminin prédominait avec un sexratio de 0,6. L’âge était renseigné pour 1182 patients, la majorité était des adultes (81 %; n = 957/1182), l’âge médian étant de 39 ans [intervalle interquartiles 26-51] (Tableau II).

**Tableau I:** Caractéristiques générales de la population d’étude

Variables	Nombre (n = 322)	%
Sexe (n = 1 256)		
masculin	469	37,3
féminin	787	62,7
Tranches d’âge (n = 1 182)		
nourrissons	30	2,6
enfants	195	16,6
adultes	817	69,2
seniors	140	11,6
Site de prélèvement (n = 1 256)		
peau	451	4
autres phanères	745	59,3
muqueuses	50	0,8
prélèvements profonds	10	35,9

Les lésions des phanères prédominaient, notamment celles des ongles des mains (24,4 %; n = 306/1256), des ongles des pieds (22,4 %; n = 281/1256) et du cuir chevelu (12,6 %; n = 158/1256). Parmi les lésions cutanées, celles de la peau glabre (15,0%; n = 189/1 256), des plantes des pieds (9,4 %; n = 118/1256), des espaces interdigito-plantaires (4,9 %; n = 62/1256) et des plis inguino-cruraux (3,8 %; n = 48/1256) étaient les plus fréquentes. La majorité des atteintes des muqueuses (n = 48/50) étaient buccales et génitales (Tableau I).

L’infection à *Candida* spp. a été diagnostiquée chez 18,4 % (n = 231/1 256) des patients. Ces candidoses prédominaient chez les femmes (75,3 %; n = 174/231) comparativement aux hommes (26,7 %; n = 57/231), chez les nourris-sons (23,3 %; n = 7/30) et les adultes (20,7 %; n = 29/140) (*p* < 0,0001). *C. albicans* était l’espèce la plus fréquemment isolée (61,0 %; n = 141/231). La répartition des espèces en fonction de l’âge a révélé que *C. albicans* était plus fréquemment isolé chez les patients âgés de moins de 18 ans (54,2 %; n = 13/24), tandis que les espèces non *albicans* prédominaient chez ceux de plus de 18 ans (58,5 %; n = 114/195). Parmi les espèces non *albicans*, *C. parapsilosis* était détecté dans 24,2 % (n = 56/231) des échantillons, *C. guiller-mondii* dans 5,2 % (n = 12/231), *C. tropicalis* dans 3,5 % (n = 8/231), *C. krusei* dans 1,7 % (n = 4/231), *C. famata* dans 1,7 % (n = 4/231). Enfin deux espèces (0,9 %) n’ont pas été identifiées. *C. rugosa*, *C. lusitaniae*, *C. magnoliae* et *C. pelliculosa* n’ont été identifiées chacune que chez un patient.

*C. albicans* était majoritairement isolé chez les hommes (82,10,7 %; n = 46/56) tandis que les es-pèces non *albicans* prédominaient chez les femmes (43,8 %; n = 74/169) (p = 0,0003). (Tableau II).

**Tableau II:** Répartition des espèces identifiées en fonction du sexe et de l’âge

Espèces identifiées*	Masculin		Féminin		Nourrissons		Enfants		Adultes		Seniors	
	n=169		n=56		n=7		n=17		n=161		n=29	
	n	%	n	%	n	%	n	%	n	%	n	%
*Candida albicans*	95	56,2	46	82,1	6	85,7	12	70,6	95	59,0	19	65,5
*Candida famata*	4	2,4	0	0	0	0	0	0	2	1,2	2	6,9
*Candida guillermondii*	11	6,5	1	1,8	0	0	0	0	11	6,8	1	3,4
*Candida krusei*	3	1,8	1	1,8	0	0	1	5,9	1	0,6	1	3,4
*Candida parapsilosis*	48	28,4	8	14,3	0	0	3	17,6	47	29,2	5	17,2
*Candida tropicalis*	8	4,7	0	0	1	14,3	1	5,9	5	3,1	1	3,4

* Âge non reporté dans les registres pour 17 patients

** p=0,01 pour la comparaison entre sexe féminin et masculin

*** p=0,25 pour la comparaison entre les tranches d'âges

Les onychomycoses et les intertrigos candido-siques représentaient la majorité des formes cliniques (82,1 %). Plus précisément, les onyxis des mains constituaient 54,5 % (n = 126/231) des atteintes candidosiques, les onychomycoses des pieds (n = 26/231) et les intertrigos interfessiers (n = 26/231), 11,3 % chacun, et les atteintes de la peau glabre 7,8 % (n = 18/231) (Tableau III).

**Tableau III:** Répartition des formes cliniques en fonction du sexe, de l’âge et des espèces identifiées

	Balanite candidosique		Candidosi buccale		Candidose cutanée		Candidose plantaire		Intertrigo interfessier		Intertrigo EID candidosique		Intertrigo inguino-crural		Intertrigo pli axillaire		Intertrigo sous-mammaire		Onyxis candidosique des mains		Onyxis candidosique des pieds		Vulvovaginite	
	(n = 2)		(n = 6)		(n = 18)		(n = 6)		(n = 1)		(n = 9)		(n = 26)		(n = 1)		(n = 1)		(n = 130)		(n = 26)		(n = 5)	
	n	%	n	%	n	%	n	%	N	%	n	%	n	%	n	%	n	%	n	%	n	%	n	%
**Sexe**																								
masculin	-	-	5	83,3	11	61,1	4	66,7	-	-	2	22,2	13	50	1	100	1	100	113	89,7	19	73,1	5	100
féminin	2	100	1	16,7	7	38,9	2	33,3	1	100	7	77,8	13	50	-	-	-	-	17	10,3	7	20,9	-	-
**Tranches d'âge**																								
nourrissons	-	-	3	50	1	5,5	-	-	-	-	-	-	3	11,5	-	-	-	-	1	0,8	-	-	-	-
enfants	-	-	-	-	5	27,8	2	33,3	-	-	1	11,1	-	-	-	-	-	-	8	6,3	-	-	1	50
adultes	2	100	3	50	12	66,7	4	66,7	1	100	6	66,7	19	73,1	1	100	1	100	95	75,4	22	91,7	1	50
seniors	-	-	-	-	-	-	-	-	-	-	2	22,2	4	15,4	-	-	-	-	22	17,5	2	8,3	-	-
**Espèces identifiées**																								
*C. albicans*	2	100	6	100	12	66,7	4	66,7	1	100	8	88,9	26	100	1	100	1	100	68	54,0	7	29,2	5	100
*C. famata*	-	-	-	-	-	-	-	-	-	-	-	-	-	-	-	-	-	-	4	3,2	-	-	-	-
*C. guillermondii*	-	-	-	-	-	-	1	16,7	-	-	-	-	-	-	-	-	-	-	7	5,6	4	16,7	-	-
*C. krusei*	-	-	-	-	1	5,6	-	-	-	-	-	-	-	-	-	-	-	-	2	1,6	1	4,2	-	-
*C. parapsilosis*	-	-	-	-	4	22,2	-	-	-	-	1	11,1	-	-	-	-	-	-	39	31	12	50	-	-
*C. tropicalis*	-	-	-	-	1	5,6	1	16,7	-	-	-	-	-	-	-	-	-	-	6	4,8	-	-	-	-

Âge non connu pour 17 patients / Deux espèces non identifiée

Les candidoses des muqueuses étaient retrouvées dans 6,1 % des cas. En fonction du sexe, les lésions cutanées candidosiques étaient plus fréquemment retrouvées chez les hommes (56,1 %; n = 32/57) tandis que les onychomycoses étaient plus fréquemment retrouvées chez les femmes (75,9 %; n = 132/174) (*p* < 0,01).

Avec l’âge, la fréquence des onychomycoses candidosiques augmentait significativement, tandis que celle des lésions cutanées candidosiques diminuait nettement. Les onychomycoses étaient davantage retrouvées chez les personnes de plus de 60 ans (82,8 %; n = 24/29) tandis que les lésions cutanées candidosiques étaient plus fréquemment observées chez les nourrissons (57,1 %; n = 4/7) (*p* < 0,01).

*C. albicans* était l’espèce la plus fréquente, quelle que soit la forme clinique. Cependant, les espèces non *albicans* colonisaient majoritairement les ongles et plus spécifiquement ceux des pieds (p < 0,01) avec principalement *C. parapsilosis*, *C. tropicalis*, *C. guillermondii* et *C. krusei* (Fig. 1).

**Figure 1:**
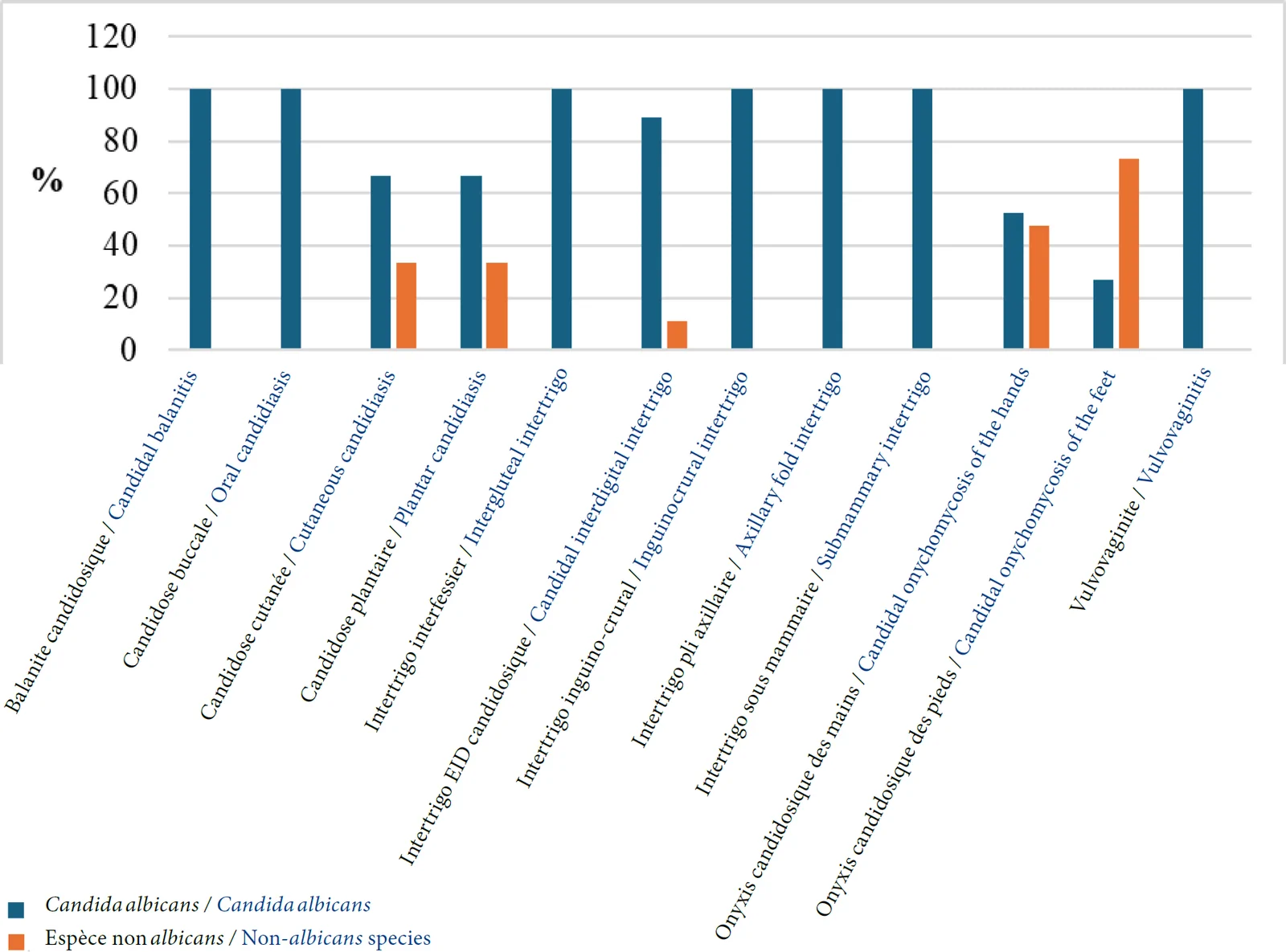
Répartition des espèces de *Candida* spp. identifiées en fonction des formes cliniques

## Discussion

Les mycoses superficielles constituent un motif fréquent de consultation en dermatologie et en mycologie. Leur prévalence varie de 20 à 25 % en zone tropicale [13]. Les atteintes dermatophytiques (notamment *Tinea capitis*), et les malassezioses sont considérées comme les plus fréquentes dans plusieurs pays d’Afrique subsaharienne [21,27], mais très peu d’études renseignent sur l’épidé-miologie et les caractéristiques des candidoses superficielles dans cette région du monde [25,28]. Au Gabon, bien que certains auteurs rapportent une fréquence non négligeable des candidoses superficielles, il existe encore peu de données sur l’épidémiologie actuelle des mycoses, tant superficielles que profondes [26]. La présente étude avait pour objectif de décrire la prévalence, le profil clinique et la diversité fongique des candidoses superficielles dans une population de patients porteurs de lésions cliniques à Libreville.

Les levures du genre *Candida* faisant partie de la flore commensale cutanéo-muqueuse, leur isolement en culture ne suffit pas, à lui seul, à affirmer une infection. Dans l’étude, les prélèvements ont été réalisés exclusivement chez des patients présentant des lésions cliniquement évocatrices, pour lesquelles une confirmation biologique était recherchée afin d’orienter la prise en charge. L’association d’une lésion compatible, de la mise en évidence de levures et surtout de pseudo-filaments à l’examen direct, et d’une culture positive interprétée dans ce contexte était consi-dérée comme suffisante pour poser le diagnostic de candidose superficielle [31]. Cette approche, centrée sur la clinique, correspond à la pratique courante dans les contextes à ressources limitées. Elle permet d’éviter l’écueil inverse consistant à assimiler toute culture positive à une infection. L’évolution favorable sous antifongiques n’a pas été recherchée du fait du contexte rétrospectif de cette étude basée sur des données de laboratoire. Cet élément relève d’un critère d’évaluation de la prise en charge et non d’un élément de validation du diagnostic, en pratique quotidienne. Les recommandations insistent par ailleurs sur la difficulté, pour certaines situations, de distinguer colonisation et infection vraie, ce qui justifie de replacer systématiquement les résultats mycologiques dans le cadre clinique [34]. Pour les atteintes unguéales, il est recommandé de confirmer la suspicion clinique d’onychomycose par des tests mycologiques (examen direct, culture et/ou histopathologie), ce qui soutient l’interprétation clinique de nos isolats [11].

Ainsi, parmi les 1256 prélèvements analysés, la fréquence des candidoses superficielles était de 18,4 %. Cette proportion est inférieure à celle observée dans une population de patients suivis entre 2013 et 2020 au DPMMT de l’USS au Gabon (66,1 %), mais supérieure à celle rapportée en Côte d’Ivoire en 2017 (7,7 %) [20,26]. Cette différence peut s’expliquer par plusieurs facteurs liés au profil des patients, au parcours de soins et à l’organisation de l’offre diagnostique. En effet, le CMO est une structure privée, où les coûts de consultation et d’examens biologiques sont plus élevés que dans les structures publiques. Seuls les patients disposant de ressources financières suffisantes ont pu accéder à une confirmation biologique systématique. Par ailleurs, durant la période 2022-2024, la fermeture temporaire du DPMMT a modifié les circuits de prise en charge. De nombreux patients présentant des lésions évocatrices de mycoses ont certainement été orientés vers d’autres praticiens, qui recouraient plus fréquemment à une prescription empirique d’antifongiques, fondée sur la clinique, sans confirmation mycologique préalable. Cette pratique a probablement contribué à une sous-représentation des cas biologiquement documentés dans notre série.

Enfin, l’administration préalable d’antifongiques avant la réalisation des prélèvements, non déclarée chez certains patients, a pu entraîner une négativation partielle des cultures, conduisant à une sousestimation de la fréquence réelle des candidoses superficielles dans la population étudiée. Globalement, les candidoses étaient plus fréquentes chez les femmes (75,3 %) que chez les hommes, contrairement aux données de Moutombi et *al*. en 2020 [26]. Les femmes sont plus susceptibles de se rendre dans un centre de soins pour une prise en charge adéquate en cas de maladie, et fréquenteraient davantage que les hommes les services d’analyses médicales. La prédominance féminine des candidoses cutanéo-muqueuses pourrait aussi être liée aux habitudes vestimentaires, esthétiques et domestiques adoptées par ces dernières.

Parmi les patients atteints, les nourrissons et les personnes âgées étaient également plus concernés. Les âges extrêmes de la vie constituent des facteurs de risque des candidoses [7].

Une analyse de la fréquence des cultures positives en fonction du site de prélèvement a montré de façon significative que les plis et les ongles étaient les sites préférentiellement colonisés par les levures du genre *Candida*. Concernant les plis interdigitaux, l’humidité retrouvée au fond des grands plis dans nos climats tropicaux est un facteur favorisant de candidose [18,26]. Il en est de même pour l’onyxis des mains, ces dernières étant plus en contact avec l’eau que les autres parties du corps. Ces résultats corroborent les données de la littérature indiquant que les candidoses super-ficielles touchent préférentiellement les zones du haut du corps, notamment la peau et ses annexes, dans les environnements tropicaux [8,23]. Ces résultats diffèrent de ceux retrouvés au Maroc par Ahaik *et al.* en 2024 où les prélèvements vaginaux, les isolats de selles et d’urines ont été classés comme principales sources cliniques. En Tunisie en revanche, les onychomycoses des pieds étaient les atteintes les plus fréquentes, suivies des onychomycoses des mains [1,4].

Une particularité notable de nos résultats est la prédominance des onychomycoses, représentant une proportion de 41,8 % pour les ongles des mains et 9,3 % pour ceux des pieds. Les infections unguéales, en particulier candidosiques, constituent une manifestation fréquente chez les patients immunocompétents des zones tropicales ou subtropicales. La forte prévalence de ces lésions peut également refléter une exposition prolongée à l’humidité, ainsi qu’un certain retard dans la prise en charge. Ce phénomène peut aussi s’expliquer par des soins de manucure répétés et agressifs entraînant des microtraumatismes de l’ongle [22,29]. L’onychomycose candidosique est souvent la manifestation clinique la plus fréquente dans les régions chaudes et humides, avec une prévalence pouvant atteindre 50 % des porteurs d’onychopathies [14].

Concernant la répartition des espèces, *C*. *albicans* demeure prédominante, retrouvée dans 61 % des isolats. Cette fréquence est supérieure à celle observée au DPMMT (41,0 %) et en Éthiopie (49,8 %), mais inférieure à celle rapportée par Seck au Sénégal (90,86 %) [26,36,37]. *C. albicans* a la capacité de mieux adhérer aux cellules épithéliales des muqueuses par rapport aux autres espèces de levures, favorisant ainsi son potentiel de colonisation et d’infection [15]. Parmi les espèces non *albicans*, *C*. *parapsilosis* (24,2 %) était la seconde levure la plus fréquente. Une étude au Togo avait révélé une fréquence de *C. parapsilosis* plus basse (5,6 %), tandis qu’une autre menée en Éthiopie révélait une fréquence plus élevée de *C*. *krusei* [37,39].

L’incidence croissante des espèces non *albicans*, souvent associée à une résistance accrue aux antifongiques, est un phénomène observé dans plusieurs régions tropicales, comme rapporté en Éthiopie [37]. La diversité des espèces soulève donc des enjeux importants pour la prise en charge thérapeutique, notamment en termes de réponse thérapeutique. Il est important de souligner que, malgré la prévalence significative de ces infections, peu d’études antérieures ont documenté la diversité des espèces de *Candida* spp. dans ce contexte, en particulier chez les populations immunocompétentes. Les présentes données contribuent à combler cette lacune en apportant des informations importantes sur la répartition des espèces et la localisation clinique superficielle de *Candida* spp. La détection de *C. parapsilosis* est particulièrement préoccupante en raison de son potentiel de résistance et de son rôle croissant dans les infections superficielles.

Ces résultats confirment que, dans les pays à ressources limitées où la majorité des lésions est traitée de manière empirique par la prescription de fluconazole, il devient crucial de disposer des données locales pour orienter la pratique clinique. La montée en fréquence des espèces non *albicans*, souvent résistantes au fluconazole (le principal antifongique prescrit dans de nombreux pays à ressources limitées), pose un défi majeur pour la prise en charge de ces infections. Par conséquent, la réalisation d’études similaires sur les candidoses superficielles et les autres mycoses endémiques au Gabon est essentielle pour éclairer les cliniciens et élaborer des stratégies de traitement adaptées, basées sur une identification précise des espèces et un suivi de leur sensibilité.

Malgré la pertinence de cette étude pour mieux comprendre la prévalence et la diversité clinique des candidoses superficielles à Libreville, quelques limites doivent être soulignées.

Tout d’abord, la nature transversale de l’étude et le recours à une collecte de données fondée sur les registres cliniques peuvent introduire des biais de sélection, notamment en limitant l’analyse aux patients ayant consulté dans une structure spécifique.

De même, le caractère rétrospectif de l’étude n’a pas permis la disponibilité de certaines variables sociodémographiques, notamment la profession des patients, ce qui a restreint l’analyse de certains facteurs socio-démographiques connus comme des déterminants ou facteurs associés aux candidoses superficielles. Néanmoins, elle a été réalisée sur une large série de patients et sur des critères cliniques et mycologiques, et apporte des données actualisées sur le profil épidémiologique et la diversité des espèces de *Candida* spp. dans notre contexte.

Une enquête multisites intégrant également la spectrométrie de masse, technique plus sensible, permettrait d’obtenir une identification plus fine des espèces, dont la diversité réelle pourrait avoir été sousestimée en l’absence de cette méthode, la diversité réelle des espèces.

## Conclusion

Notre étude a mis en évidence une fréquence non négligeable des candidoses comme étiologie des lésions cutanéomuqueuses. Les onychomycoses et les intertrigos candidosiques constituent les formes cliniques prédominantes. Bien que *C. albicans* soit majoritaire, une évolution de l’épidémiologie vers les espèces non *albicans* est observée, ce qui souligne la nécessité d’une prise en charge adaptée au contexte local et d’une confirmation biologique des suspicions cliniques.

## Remerciements

Nous remercions le personnel du laboratoire du CMO qui a accepté de participer à l’étude, ainsi que le personnel du DPMMT-USS. Nos remer-ciements vont à toute l’équipe ayant rendu ce travail possible.

## Financement

Ce projet n’a bénéficié d’aucun financement, qu’il soit public, privé, institutionnel ou industriel. Les travaux menés dans le cadre de ce projet ont été réalisés sans soutien financier externe, sub-vention, bourse ou contrat de recherche

## Contributions des auteurs et autrices

Coëlla Joyce MIHINDOU: analyse des données, préparation et rédaction du manuscrit

Reinne MOUTONGO MOUANDZA: dévelop-pement et conception des méthodes

Bridy Chesly MOUTOMBI DITOMBI: conduc-tion du processus de recherche, investigation et collection des données

Ardin Dimitri MOUSSAVOU MABICKA : re-cherche de la littérature

Charleine MANOMBA BOULINGUI: conduc-tion du processus de recherche, investigation et collection des données

Christian MAYANDZA: collection des données Roger Hadry SIBI MATOTOU : développement et conception des méthodes

Noé Patrick M’BONDOUKWE: management et supervision des activités de recherche Magalie ESSOMEYO NGUE MEBALE: collection des données

Denise Patricia MAWILI MBOUMBA : management et supervision des activités de recherche Marielle Karine

BOUYOU AKOTET: management et supervision des activités de recherche, no-tamment celle de cette étude ainsi que validation des résultats, relecture et révision du manuscrit.

## Déclaration de liens d’intérêts

Aucun lien d’intérêt n’a été déclaré.
