# Supplementary material for: Suspected superficial candidiasis in Libreville: clinical profiles and distribution of Candida spp. (2022–2024)
Source: Med Trop Sante Int. 2026 Jul 27;6(3):mtsi.v6i3.2026.742. [Article in French] doi: 10.48327/mtsi.v6i3.2026.742 (PMC13697909; doi:10.48327/mtsi.v6i3.2026.742)
Supplement: Related texts

# Research article: Suspected superficial candidiasis in Libreville: clinical profiles and distribution of *Candida* spp. (2022–2024)

## Introduction

The increasing prevalence of infections caused by human fungal pathogens is a growing and underestimated threat, largely due to the increased resistance to antifungal treatments. Superficial fungal infections are among the most common conditions worldwide, affecting approximately 20–25% of the population [9,17,32]. In addition to dermatophytoses and malassezioses, clinicians most frequently consider superficial candidiasis to be the cause [33].

*Candida* yeasts are part of the human commensal flora and can colonize the mucous membranes of the genital, urinary, respiratory and gastrointestinal tracts, as well as the oral cavity, nails, scalp and skin [2, 10]. *Candida albicans* is the most frequently isolated species, but *C.* non-*al-bicans* species such as *C. parapsilosis*, *C. krusei* and *C. glabrata* are increasingly responsible for superficial infections [6,16,19,28].

In Gabon, superficial clinical lesions are the most common presentation in samples submitted to the mycology laboratory at the Department of Parasitology, Mycology, and Tropical Medicine (DPMMT) at the Faculty of Medicine of the University of Health Sciences (USS). The DPMMT is the reference laboratory for the clinical and biological diagnosis of fungal infections in Gabon. However, the medical community has limited information regarding the clinical spectrum and diversity of causative species, and therapeutic options remain limited. Indeed, high prevalence of resistance to commonly used antifungals, such as fluconazole, econazole, and amphotericin B, has been observed in *C. albicans* and *C.* non-*albicans* [24,30]. Empirically prescribing antifungals, particularly fluconazole (the most readily available drug in Gabon), to immunocompromised patients to prevent opportunistic fungal infections or to immunocompetent patients for presumptive curative treatment is a risk factor for the emergence of resistance to this drug.

A recent analysis of data collected by the DPMMT from people living with HIV (PLHIV) revealed a shift in the fungal flora profile of gastrointestinal candidiasis in Libreville [26,30,38]. However, the epidemiology of superficial candidiasis remains poorly understood. Generating information on these common conditions would enable the development of decision-making algorithms and appropriate management protocols. This information could also serve as an advocacy tool for policymakers, improving access to other azole antifungals.

This study aimed to describe the prevalence, clinical profile, and fungal diversity of superfi-cial candidiasis in patients with clinical lesions in Libreville.

## Methods

This retrospective descriptive study was conducted at the Oloumi Medical Clinic (CMO) in Libreville by the DPMMT team of the USS.

Data from patient registries of individuals who underwent comprehensive mycological examinations for lesions suspected to be superficial fungal infections between 2022 and 2024 were used for the study. The following information was recorded on a data collection form and analyzed within the DPMMT: presence of a suspected fungal lesion, lesion site, mycological examination result, age, and sex. Patients were divided into four groups based on age: infants under two years of age, children aged two to 17 years, adults aged 18 to 59 years, and seniors aged 60 years or older.

Samples were collected under aseptic conditions. Skin lesions were sampled by scraping scales from the edges of the lesions. Mucous membrane lesions were sampled by swabbing with a sterile swab or scraping with a sterile scalpel. Nail lesions were sampled by scraping scales from the edges of the lesions. Scalp lesions were sampled by collecting scales from the scalp and affected body or scalp hair. Vaginal or urine samples were collected when candidiasis of the genital mucous membranes was suspected.

Direct microscopic examination was performed with a drop of 30% potassium hydroxide solution between a slide and a coverslip to clarify thick preparations, such as those from skin appendages. A physiological saline solution was used for swabs. Cultures were performed by inoculating standard mycology media (Sabouraud-chloramphenicol and Sabouraud-chloramphenicol-actidione) and incubating them at 37°C. The cultures were checked after 24 hours and daily for eight days. A culture was considered positive if creamy, domed colonies ranging in color from white to cream were present. For mucosal swabs, the presence of at least 10 colonies of *C. albicans* confirmed the diagnosis of candidiasis.

Identification and differentiation between *C. albicans* and *C. dubliniensis* were performed using Candida ID2 chromogenic media (Biomérieux) in accordance with the manufacturer’s recom-mendations and the procedures described in the literature [12]. Identification was supplemented by an auxanogram using API 20 C AUX strips [3,35]. Superficial candidiasis was defined by a combination of clinical and mycological findings based on the following: (i) the presence of a cutaneous-mucosal lesion suggestive of superficial mycosis, (ii) a positive direct mycological examination demonstrating budding yeasts and/or pseudofilaments associated with a local inflammatory reaction evidenced by the presence of ≥10 leukocytes per field, and/or (iii) a positive culture with growth of ≥10 *Candida* spp. colonies.

In this study, candidiasis was diagnosed based exclusively on clinical and mycological criteria. In accordance with international recommendations [5,34], laboratory confirmation rather than the response to treatment was the determining factor for the indication of antifungal treatment. Response to treatment was part of the evaluation of care and was not used as a criterion for diagnostic confirmation.

*Candida* spp. colonization was defined as its isolation in culture from asymptomatic subjects in the absence of clinical signs or microscopic criteria suggestive of invasion [5,31,34].

The data were entered into an Excel file and analyzed using StatView 5.0 software. Qualitative data were presented as proportions and compared using the chi-square test or Fisher’s exact test. The significance threshold was set at p < 0.05.

## Results

Data from 1,256 patients who underwent mycological examinations for suspected cutaneous-mucosal mycoses were analyzed. Females predominated, with a sex ratio of 0.6. Age was recorded for 1,182 patients. The majority were adults (81%; n = 957/1,182), with a median age of 39 years [interquartile range 26–51] (Table I). Lesions of the appendages were predominant, particularly those of the fingernails (24.4%; n = 306/1,256), toenails (22.4%; n = 281/1,256), and scalp (12.6%; n = 158/1,256). The most common skin lesions were those involving hairless skin (15.0%; n = 189/1,256), the soles of the feet (9.4%; n = 118/1,256), the interdigital spaces of the feet (4.9%; n = 62/1,256), and the inguinal-femoral folds (3.8%; n = 48/1,256). The majority of mucosal lesions (n = 48/50) were oral and genital (Table I). *Candida* spp. infection was diagnosed in 18.4% (n = 231/1,256) of patients. *Candida* infections were more prevalent in women (75.3%; 174/231) than in men (26.7%; 57/231) and in infants (23.3%; 7/30) than in adults (20.7%; 29/140) (p < 0.0001). *C. albicans* was the most frequently isolated species (61.0%; n = 141/231).

**Table I:** General characteristics of the study population

Variable	Headcount (n = 322)	%
Gender (n = 1,256)		
male	469	37,3
female	787	62,7
Age groups (n = 1,182)		
infants	30	2,6
children	195	16,6
adults	817	69,2
seniors	140	11,6
Sample collection site (n = 1,256)		
skin	451	4
other appendages	745	59,3
mucous membranes	50	0,8
deep samples	10	35,9

The age-based distribution of species revealed that *C. albicans* was more frequently isolated in patients under 18 years of age (54.2%; n = 13/24), whereas non-*albicans* species predominated in patients over 18 years of age (58.5%; n = 114/195). Of the non-*albicans* species, *C. parapsilosis* was present in 24.2% (56/231) of samples, *C. guillier-mondii* in 5.2% (12/231), *C. tropicalis* in 3.5% (8/231), *C. krusei* in 1.7% (4/231), and *C. famata* in 1.7% (4/231). Finally, two species (0.9%) were not identified. *C. rugosa, C. lusitaniae, C. mag-noliae*, and *C. pelliculosa* were each identified in one patient only.

*C. albicans* predominated in men (82.1%; n = 46/56), while non-*albicans* species predominated in women (43.8%; n = 74/169) (p = 0.0003) (Table II).

**Table II:** Distribution of identified species by sex and age

Species identified*	Male		Female		Infants		Children		Adults		Seniors	
	n=169		n=56		n=7		n=17		n=161		n=29	
	n	%	n	%	n	%	n	%	n	%	n	%
*Candida albicans*	95	56,2	46	82,1	6	85,7	12	70,6	95	59,0	19	65,5
*Candida famata*	4	2,4	0	0	0	0	0	0	2	1,2	2	6,9
*Candida guillermondii*	11	6,5	1	1,8	0	0	0	0	11	6,8	1	3,4
*Candida krusei*	3	1,8	1	1,8	0	0	1	5,9	1	0,6	1	3,4
*Candida parapsilosis*	48	28,4	8	14,3	0	0	3	17,6	47	29,2	5	17,2
*Candida tropicalis*	8	4,7	0	0	1	14,3	1	5,9	5	3,1	1	3,4

* Age not recorded in the records for 17 patients

** p=0.01 for the comparison between females and males

*** p=0.25 for the comparison between age groups

Onychomycoses and candidal intertrigo accounted for most clinical forms (82.1%). Specifically, onychomycosis of the hands accounted for 54.5% (n = 126/231) of candidal infections; onychomycosis of the feet and intergluteal intertrigo each accounted for 11.3% (n = 26/231); and lesions of the glabrous skin accounted for 7.8% (n = 18/231) (Table III). Mucosal candidiasis was found in 6.1% of cases. Cutaneous candidiasis lesions were more frequently found in men (56.1%; n = 32/57), while onychomycosis was more frequently found in women (75.9%; n = 132/174) (p < 0.01).

**Table III:** Distribution of clinical forms by sex, age, and identified species

	Candidal balanitis		Oral candidiasis		Cutaneous candidiasis		Plantar candidiasis		Intertrigo of the buttocks		Candidal interdigital intertrigo		Inguinal-crural intertrigo		Axillary intertrigo		Submammary intertrigo		Candida-induced onychomycosis of the hands		Candida-induced onychomycosis of the feet		Vulvovaginitis	
	(n = 2)		(n = 6)		(n = 18)		(n = 6)		(n = 1)		(n = 9)		(n = 26)		(n = 1)		(n = 1)		(n = 130)		(n = 26)		(n = 5)	
	n	%	n	%	n	%	n	%	N	%	n	%	n	%	n	%	n	%	n	%	n	%	n	%
Gender																								
male	-	-	5	83,3	11	61,1	4	66,7	-	-	2	22,2	13	50	1	100	1	100	113	89,7	19	73,1	5	100
female	2	100	1	16,7	7	38,9	2	33,3	1	100	7	77,8	13	50	-	-	-	-	17	10,3	7	20,9	-	-
Age groups																								
infants	-	-	3	50	1	5,5	-	-	-	-	-	-	3	11,5	-	-	-	-	1	0,8	-	-	-	-
children	-	-	-	-	5	27,8	2	33,3	-	-	1	11,1	-	-	-	-	-	-	8	6,3	-	-	1	50
adults	2	100	3	50	12	66,7	4	66,7	1	100	6	66,7	19	73,1	1	100	1	100	95	75,4	22	91,7	1	50
seniors	-	-	-	-	-	-	-	-	-	-	2	22,2	4	15,4	-	-	-	-	22	17,5	2	8,3	-	-
Identified species																								
*C. albicans*	2	100	6	100	12	66,7	4	66,7	1	100	8	88,9	26	100	1	100	1	100	68	54,0	7	29,2	5	100
*C. famata*	-	-	-	-	-	-	-	-	-	-	-	-	-	-	-	-	-	-	4	3,2	-	-	-	-
*C. guillermondii*	-	-	-	-	-	-	1	16,7	-	-	-	-	-	-	-	-	-	-	7	5,6	4	16,7	-	-
*C. krusei*	-	-	-	-	1	5,6	-	-	-	-	-	-	-	-	-	-	-	-	2	1,6	1	4,2	-	-
*C. parapsilosis*	-	-	-	-	4	22,2	-	-	-	-	1	11,1	-	-	-	-	-	-	39	31	12	50	-	-
*C. tropicalis*	-	-	-	-	1	5,6	1	16,7	-	-	-	-	-	-	-	-	-	-	6	4,8	-	-	-	-

Age unknown for 17 patients / Two unidentified species

The frequency of candidal onychomycosis in-creased significantly with increasing age, while the frequency of candidal skin lesions decreased markedly. Onychomycosis was more prevalent in individuals over 60 years of age (82.8%; n = 24/29), while candidal skin lesions were more prevalent in infants (57.1%; n = 4/7) (p < 0.01).

*C. albicans* was the most common species regard-less of clinical presentation. However, non-*albicans* species predominantly colonized the nails, particularly the nails on the feet (p < 0.01), with *C. parapsilosis, C. tropicalis, C. guillermondii*, and *C. krusei* being the most common (Fig. 1).

**Figure 1:** Distribution of identified *Candida* spp. species by clinical presentation

## Discussion

Superficial mycoses are a common reason for consultation in dermatology and mycology. In tropical regions, their prevalence ranges from 20 to 25% [13]. Dermatophyte infections, particularly *Tinea capitis*, and *Malassezia* infections are the most prevalent in several sub-Saharan African countries [21,27], but few studies provide information on the epidemiology and characteristics of superficial candidiasis in this region [25,28]. In Gabon, some authors report a significant prevalence of superficial candidiasis; however, there is still little data on the current epidemiology of fungal infections, both superficial and deep [26]. This study aimed to describe the prevalence, clinical profile, and fungal diversity of superficial candidiasis in patients with clinical lesions in Libreville.

Since *Candida* yeasts are part of the commensal skin and mucosal flora, isolating them in culture is insufficient to confirm an infection. For this reason, samples were collected exclusively from patients presenting clinically suggestive lesions for whom biological confirmation was sought to guide management. In this context, the combination of a compatible lesion, the detection of yeasts and pseudofilaments on direct examination, and a positive culture was considered sufficient to establish a diagnosis of superficial candidiasis [31]. This clinically centered approach corresponds to standard practice in settings with limited resources. It helps avoid equating any positive culture with an infection. Due to the retrospective nature of this study based on laboratory data, favorable response to antifungal therapy was not assessed. This aspect relates to a criterion for evaluating management rather than a criterion for validating a diagnosis in daily practice. The recommendations also emphasize the difficulty of distinguishing between colonization and true infection in certain situations, which justifies placing mycological results within the clinical context systematically [34]. For nail infections, it is recommended that clinical suspicion of onychomycosis be confirmed through mycological testing, such as direct examination, culture, and/ or histopathology. This testing supports the clinical interpretation of our isolates [11].

Thus, the prevalence of superficial candidiasis was 18.4% among the 1,256 samples analyzed. This proportion is lower than that observed in a patient population monitored from 2013 to 2020 at the DPMMT of the USS in Gabon (66.1%) but higher than that reported in Côte d’Ivoire in 2017 (7.7%) [20,26]. These differences can be explained by various factors related to patient profiles, the care pathway, and the organization of diagnostic services. The CMO is a private facility where consultation and laboratory test costs are higher than in public facilities. Only patients with sufficient financial resources could access systematic laboratory confirmation. Furthermore, the temporary closure of the DPMMT during the 2022–2024 period altered care pathways. Many patients presenting with lesions suggestive of fungal infections were likely referred to other practitioners who more frequently prescribed antifungals empirically based on clinical presentation without prior mycological confirmation. This practice likely contributed to the underrepresentation of biologically documented cases in our study.

Additionally, prior administration of antifungals before specimen collection, which was not reported for some patients, may have led to partial negative culture results. This resulted in an under-estimation of the actual prevalence of superficial candidiasis in the study population.

Overall, candidiasis was more prevalent in women (75.3%) than in men, contrary to Moutombi et al.’s 2020 findings [26]. Women are more likely than men to seek care at a healthcare facility for the proper management of illness and are more frequent users of laboratory services. The higher prevalence of cutaneous and mucosal candidiasis among women may also be linked to their clothing, grooming, and household habits.

Among affected patients, infants and the elderly were also more commonly affected. The extreme ages of life are risk factors for candidiasis [7].

A significant analysis of the frequency of positive cultures by sampling site showed that skin folds and nails were the sites most frequently colonized by *Candida* yeasts. Regarding interdigital folds, the moisture at the base of large skin folds in tropical climates favors candidiasis [18,26]. The same applies to onychomycosis of the hands because the hands are in more frequent contact with water than other parts of the body. These results corroborate literature indicating that superficial candidiasis preferentially affects the upper body, particularly the skin and its appendages, in tropical environments [8,23]. However, these results differ from those found in Morocco by Ahaik *et al.* in 2024, where vaginal swabs, stool, and urine isolates were classified as the primary clinical sources. In Tunisia, however, onychomycosis of the feet was the most common infection, followed by onychomycosis of the hands [1,4].

Notably, onychomycosis accounted for 41.8% of fingernail infections and 9.3% of toenail infections in our study. Nail infections, particularly those caused by *Candida*, are common in immuno-competent patients in tropical or subtropical regions. The high prevalence of these lesions may reflect prolonged exposure to humidity and delayed treatment. This phenomenon may also be explained by repeated and aggressive manicure procedures that lead to microtrauma of the nail [22,29]. *Candida* onychomycosis is often the most prevalent clinical manifestation in hot, humid regions, affecting up to 50% of patients with onychopathies [14].

Regarding species distribution, *C. albicans* remains predominant, accounting for 61% of isolates. This frequency is higher than that observed at the DPMMT (41.0%) and in Ethiopia (49.8%), though it is lower than the frequency reported by Seck in Senegal (90.86%) [26,36,37]. *C. albicans* has a greater ability to adhere to mucosal epithelial cells than other yeast species, thereby enhancing its potential for colonization and infection [15]. Among non-*albicans* species, *C. parapsilosis* (24.2%) was the second most prevalent yeast. One study in Togo revealed a lower frequency of *C. parapsilosis* (5.6%), while another study in Ethiopia revealed a higher frequency of *C. krusei* [37,39].

The increasing prevalence of non-*albicans* species, which are often associated with increased resistance to antifungal agents, has been observed in several tropical regions, including Ethiopia [37]. This raises significant challenges for clinical management, particularly in terms of treatment response. Despite the significant prevalence of these infections, few previous studies have documented *Candida* spp. diversity in this context, particularly among immunocompetent populations. The present data help fill this gap by providing important information on the distribution of *Candida* spp. and their superficial clinical localization. The detection of *C. parapsilosis* is of particular concern due to its potential for resistance and its growing role in superficial infections.

These results confirm that local data are crucial to guide clinical practice in resource-limited countries where the majority of lesions are treated empirically with fluconazole. The rising prevalence of non-*albicans* species, which are often resistant to fluconazole (the primary antifungal prescribed in many resource-limited countries), poses a significant challenge to managing these infections. Consequently, conducting similar studies on superficial candidiasis and other endemic mycoses in Gabon is essential to inform clinicians and develop appropriate treatment strategies based on accurate species identification and monitoring of susceptibility.

Despite this study’s relevance to understanding the prevalence and clinical diversity of superficial candidiasis in Libreville, several limitations should be noted.

First, the cross-sectional nature of the study and the use of data collection based on clinical records may introduce selection bias, particularly by limiting the analysis to patients who sought care at a specific facility.

Similarly, the retrospective nature of the study did not allow for the availability of certain sociodemographic variables, notably patients’ occupations, which limited the analysis of certain sociodemographic factors known to be determinants or factors associated with superficial candidiasis. Nevertheless, it was conducted on a large series of patients using clinical and mycological criteria and provides up-to-date data on the epidemiological profile and species diversity of *Candida* spp. in our setting.

A multicenter study incorporating mass spectrometry, a more sensitive technique, would allow for a more precise identification of species, the true diversity of which may have been underestimated in the absence of this method.

## Conclusion

Our study highlighted a significant frequency of candidiasis as the etiology of cutaneous and mucosal lesions. Onychomycoses and candidal intertrigo are the predominant clinical forms. Although *C. albicans* is the most common, a shift in the epidemiology toward non-*albicans* species is observed, underscoring the need for management tailored to the local context and for biological confirmation of clinical suspicions.

## Acknowledgments

We thank the staff of the CMO laboratory who agreed to participate in the study, as well as the staff of the DPMMT-USS. Our thanks go to the entire team that made this work possible.

## Funding

This project did not receive any funding, wheth-er public, private, institutional, or industrial. All work conducted as part of this project was carried out without external financial support, grants, scholarships, or research contracts

## Authors’ contributions

Coëlla Joyce MIHINDOU: Data analysis, man-uscript preparation and drafting

Reinne MOUTONGO MOUANDZA: Methodology development and design

Bridy Chesly MOUTOMBI DITOMBI: Research process, investigation, and data collection Ardin Dimitri MOUSSAVOU MABICKA: Literature review

Charleine MANOMBA BOULNGUI: Research process, investigation, and data collection Christian MAYANDZA: Data collection

Roger Hadry SIBI MATOTOU: Methodology development and design

Noé Patrick MBONDOUKWE: Research activity management and supervision

Magalie ESSOMEYO NGUE MEBALE: Data collection

Denise Patricia MAWILI MBOUMBA: Research activity management and supervision Marielle Karine BOUYOU AKOTET: Research activity management and supervision, including for this study; validation of results; proofreading; and revision of the manuscript.

## Conflicts of interest

The authors declare no conflicts of interest.
